# Evaluation of the Free Radical Scavenging Activities of Ellagic Acid and Ellagic Acid Peracetate by EPR Spectrometry †

**DOI:** 10.3390/molecules26164800

**Published:** 2021-08-08

**Authors:** Ajit Kumar, Preeti Kaushik, Sandra Incerpi, Jens Z. Pedersen, Sanjay Goel, Ashok K. Prasad, Vishwajeet Rohil, Virinder S. Parmar, Luciano Saso, Christophe Len

**Affiliations:** 1 Department of Chemistry, SRM University, Delhi-NCR, Haryana, 39, RGEC, Sonepat 131 029, India; ajit.k@srmuniversity.ac.in (A.K.); preeti97kaushik@gmail.com (P.K.); 2 Department of Biochemistry, V. P. Chest Institute, University of Delhi, Delhi 110 007, India; sanjaygoelvpci@rediffmail.com (S.G.); vishrohil@gmail.com (V.R.); 3 Department of Sciences, University of Rome “Roma Tre”, 00146 Rome, Italy; sandra.incerpi@uniroma3.it; 4 Department of Biology, University of Rome “Tor Vergata”, Via della Ricerca, Scientifica 1, 00133 Rome, Italy; j.z.pedersen@gmail.com; 5 Bioorganic Laboratory, Department of Chemistry, University of Delhi, Delhi 110 007, India; ashokenzyme@gmail.com (A.K.P.); virparmar@gmail.com (V.S.P.); 6 Department of Chemistry and Environmental Science, Medgar Evers College, The City University of New York, 1638 Bedford Avenue, Brooklyn, NY 11225, USA; 7 Department of Physiology and Pharmacology “Vittorio Erspamer”, Sapienza University, P. le. Aldo Moro 5, 00185 Rome, Italy; luciano.saso@uniroma1.it; 8 Institute of Chemistry for Life and Health Sciences, Chimie ParisTech, PSL Research University, CNRS, UMR8060, 11 rue Pierre et Marie Curie, F-75005 Paris, France

**Keywords:** free radical, ellagic acid, ellagic acid peracetate, electron paramagnetic resonance, cytotoxicity, L-6 myoblasts

## Abstract

The purpose of this study was to examine the free radical scavenging and antioxidant activities of ellagic acid (EA) and ellagic acid peracetate (EAPA) by measuring their reactions with the radicals, 2,2-diphenyl-1-picrylhydrazyl and galvinoxyl using EPR spectroscopy. We have also evaluated the influence of EA and EAPA on the ROS production in L-6 myoblasts and rat liver microsomal lipid peroxidation catalyzed by NADPH. The results obtained clearly indicated that EA has tremendous ability to scavenge free radicals, even at concentration of 1 µM. Interestingly even in the absence of esterase, EAPA, the acetylated product of EA, was also found to be a good scavenger but at a relatively slower rate. Kinetic studies revealed that both EA and EAPA have ability to scavenge free radicals at the concentrations of 1 µM over extended periods of time. In cellular systems, EA and EAPA were found to have similar potentials for the inhibition of ROS production in L-6 myoblasts and NADPH-dependent catalyzed microsomal lipid peroxidation.

## Highlights

- EA and EAPA were found to be very good radical scavengers even at 1 μM.
- Rate of radical scavenging reaction by EAPA was found to be slower than that of EA in scavenging the radical, as measured by EPR.
- EA and EAPA decreased ROS production in L-6 myoblasts even at very low concentrations.
- They were both found to inhibit the NADPH catalyzed microsomal lipid peroxidation.

## 1. Introduction

Reactive oxygen species (ROS) are well-known mediators of intracellular signaling cascades. It is found that excessive production of ROS inside cells is due to exposure to several endogenous and exogenous substances, which causes damage to many important biomolecules that have been implicated in several human diseases [1]. Endogenous antioxidants are keeping pro-oxidant levels in check, but in the conditions of diseases, the balance is shifted in favor of pro-oxidants, leading to oxidative stress. Exogenous dietary antioxidants have ability to scavenge free radicals that may thus have potential to prevent various diseases [2]. Ellagic acid (EA) is a naturally occurring phenolic lactone compound found in the form of hydrolyzable tannins called ellagitannins as the structural components of plant cell walls and cell membranes. It is also observed that EA is found in many berries such as strawberries, raspberries, cranberries and grapes in high concentrations [3]. EA is also found in walnuts, pecans, grapes [4] and in distilled beverages [5]. Various studies have reported that EA also possesses antimutagenic, antioxidant, antimicrobial and anti-inflammatory activities in bacterial and mammalian systems [6,7].

Upon oral administration, EA inhibits carbon tetrachloride induced hepatotoxicity in vitro and in vivo [8]. EA induces G1 arrest in the cell cycle, inhibits overall cell growth, and apoptosis in tumor cells [9]. It also prevents esophageal cancer both at the initiation and promotion stages in animals [10]. It decreases arylamine *N*-acetyltransferase activity and DNA adduct formation in human bladder tumor cell lines T24 and TSGH 8301 [11]. EA inhibits several ROS and NOS, such as hydroxyl, peroxyl and nitrogen dioxide radicals and γ−radiation induced lipid peroxidation [12]. In one report, EA was found to prevent cisplatin-induced oxidative stress in liver [13]. It was also found that EA inhibits heart tissue of rats and fibrotic markers (MMPs and TIMPs) during alcohol-induced hepatotoxicity [14]. As evident from the above discussion, a lot of research work has been carried out on different physiological and pharmacological aspects of EA. However, detailed studies using electron paramagnetic resonance (EPR) spectroscopy on the free radical scavenging activity of EA, especially that of its acetylated product, viz. EAPA, are not known. In this communication, the antioxidant activities of EA and EAPA are reported in terms of their abilities to scavenge free radicals and also to inhibit the cumene hydroperoxide generated free radical activities in L-6 myoblasts and in rat liver microsomal lipid peroxidation catalyzed by NADPH.

## 2. Results and Discussion

### 2.1. Radical Scavenging Activities of EA and EAPA Measured by EPR

**Figure 1:**
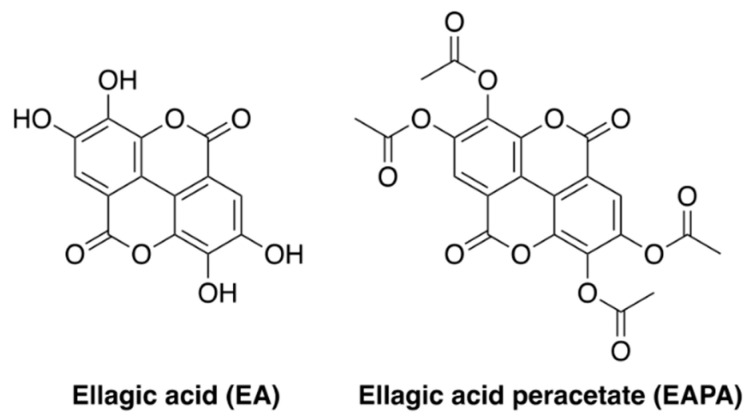
Structures of ellagic acid (EA) and the corresponding peracetate (EAPA).

The capacity to scavenge free radicals was tested with a very simple assay system. EA and EAPA (Figure 1) were mixed separately with a radical and the reaction followed by EPR spectroscopy [15,16,17]. A systematic screening of EA and EAPA was made at different concentrations in ethanol in the presence of 100 micromolar DPPH. Some measurements were also made with lower concentrations, or using another radical, galvinoxyl. It was observed that the EPR signals of DPPH and galvinoxyl were directly proportional to the concentration of the radical, but when these radicals were reduced by the antioxidant, the spectrum disappeared. It was observed that both EA and EAPA have ability to scavenge the free radical DPPH (Table 1) and galvinoxyl (Table 2) in a dose-dependent manner. It was found that EA has tremendous ability to scavenge the free radicals, while EAPA scavenges free radicals at a relatively slower rate. To measure appropriate concentration of EA, it was possible to detect the spectrum of the corresponding coumaryl radical which superimposed well on the galvinoxyl or DPPH spectrum immediately after mixing the sample. It was observed that all the coumaryl radicals were too reactive to allow characterization and disappeared within seconds (data not shown). The time dependent kinetics for the EA and EAPA are presented in Table 1 and Table 2. At a suitable concentration, EA was so active that DPPH and galvinoxyl EPR signals disappeared immediately even before the measurement of the spectrum. Acetylated product of EA, i.e., EAPA, was also found to be active in scavenging of free radicals, such as DPPH and galvinoxyl, but at a slower rate. Therefore, both EA and EAPA were tested at lower concentrations (1–2.5 μM), which allowed detection of the radical level after a prolonged time. It is pertinent to note that 1 μM of EA and EAPA scavenged the target radicals even after 48 h at a very slow rate.

### 2.2. Radical Scavenging Activities of EA and EAPA by Spectrophotometer

The scavenging activities by EA and EAPA of the free radicals, galvinoxyl, a phenoxyl-type radical, and DPPH, a nitrogen-based radical, were confirmed using EPR. Galvinoxyl and DPPH are strongly colored compounds, so the antioxidant reactions could also be measured spectrophotometrically. For this purpose, the same concentration of DPPH radical was used, which was used for EPR experiments. The 1,1-diphenyl-2-picrylhydrazyl (DPPH) assay is a well-known method for the estimation of antioxidant activities [18,19] of different compounds. In the present work, we also determined the free radical scavenging activities of ellagic acid (EA) and its acetylated product ellagic acid peracetate (EAPA) by following the absorbance of DPPH radical at 517 nm. The decrease in the absorbance was monitored at different concentrations of the EA and EAPA, from which the IC_50_ value was determined. Thus, at a DPPH concentration of 100 μM, the IC_50_ values for EA and EAPA were found to be 17 ± 4 μM and 32 ± 6 μM, respectively. It was also observed that the products formed in the reactions are strongly colored and absorb in the same spectral region, and making it complicated to determine the kinetics. The advantages of the EPR assay are that only radicals are observed, which can be detected at very low concentrations [20]. The mechanism of hydrogen transfer from polyphenols is very well established. Free radical scavenging activity of EA via the electron-oxidation mechanism has also been proposed in the recent studies [21], it is reported that the most acidic C-3 OH group of EA is the active site for radical inactivation. This observation is based on the calculation of the density functional theory and the semiempirical PM6 Method [22]. The free radical scavenging activity of EAPA is not through the conventional mechanisms of hydrogen transfer from polyphenols. It was also observed that even one and two micromolar concentration of EA and EAPA scavenged the radicals slowly over time.

**Scheme 1:**
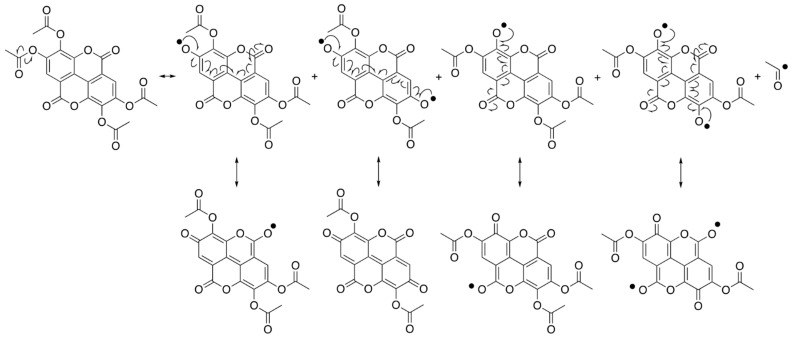
Flow Chart showing the extensive resonance stabilization of the bis-coumarin phenoxyl radical obtained from EAPA.

In our previous studies, it has been shown that 7,8-dihydroxy-4-methylcoumarin (DHMC) and its acetylated product, 7,8-diacetoxy-4-methylcoumarin (DAMC) have abilities to scavenge free radicals in a solvent system and inhibit membrane lipid peroxidation. The antioxidant properties of the polyphenolic compounds are well-known, pulse radiolysis studies reveal that DAMC and DHMC react with the system generating azide radicals and the resulting transient spectra in both cases demonstrate a peak at 410 nm, characteristic of the phenoxyl radical. The rate constants for the formation of phenoxyl radicals from DHMC and DAMC were: 34 × 10^8^ M^−1^ s^−1^ and 6.2 × 10^8^ M^−1^ s^−1^, respectively. It is proposed that the free radical mediated oxidation of DAMC initially produces a radical cation that loses an acetyl carbocation to yield the phenoxyl radical [23,24]. Both EA and EAPA are condensed bis-coumarins, which have four hydroxy and acetoxy groups, respectively. Hence, it is possible to conclude that the mechanism of the antioxidant action of both EA and EAPA (Scheme 1) follows the pathway similar to that of DHMC and DAMC involving the formation of an extensively resonance stabilized bis-coumarin-phenoxyl radical (Scheme 1). The rate constants of DHMC and DAMC also justify the explanation of lower rate of EAPA as compared to its precursor EA.

### 2.3. Antioxidant Activities of EA and EAPA

The results confirm that EA, which has two *ortho*-dihydroxy group moieties, is a very good radical scavenger; its acetylated product EAPA also has radical scavenging activity with slower rate as compared to its precursor EA. Many authors have reported that two or more hydroxy groups at the *ortho* positions is more favorable than those having the hydroxy groups at the *meta* position for coumarins [23,24,25]. EA is a well-known natural compound which possesses excellent pharmacological and physiological properties, such as anticancer and antioxidant, anti-inflammatory, etc. An amazing result was the strong scavenging activity of EAPA, which has two ortho-acetoxy groups instead of two *ortho* hydroxy groups as in EA. Although EAPA was less effective than EA, this behavior has been noticed before the formation of deactivated compounds through the activity of esterases in biological systems, or to the interaction with lipid ketene structures [23,24]. However, in the EPR experiments, the samples contained only the EAPA, the target radical, ethanol and DMSO, leaving little doubt that the reaction proceeds via one-electron reduction of the radical, excluding even the possibility of a hydrogen-atom-transfer mechanism. EAPA displays diverse biological affects that seem to be independent of its antioxidant activity, i.e., it inhibits binding of aflatoxin B_1_ to DNA in vitro and aflatoxin B_1_ induced micronuclei formation in rat bone marrow and lung cells in vivo, both of these seem to be responsible for its antimutagenic properties [26]. EAPA was found to have superior activity than the corresponding polyphenol EA [26], probably due to protein acetylation catalyzed by Calreticulin Transacetylase [27,28,29]; EA is not a substrate for Calreticulin Transacetylase but is actually the product of this biochemical reaction on EAPA. In this case, it can be concluded that there is a clear separation of enzymatic and antioxidant effects, which shows that all these diverse activities may involve both antioxidant and non-antioxidant mechanisms.

### 2.4. Intracellular Antioxidant Activities of EA and EAPA

**Figure 2:**
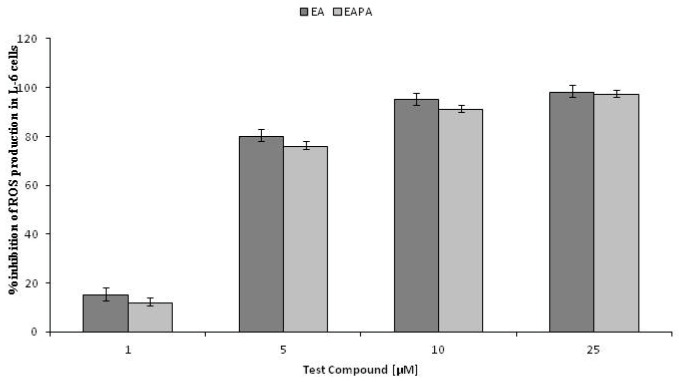
Influence of EA and EAPA on the levels of intracellular ROS formed in L-6 myoblasts after exposure to cumene hydroperoxide. Data represent mean ± S.D. from three independent experiments performed in triplicates. There were no significant differences in the activities between EA and EAPA in all the cases.

Efforts were also made to examine whether the antioxidant effects of EA and EAPA could be exhibited directly in cells. The standard assay method based on fluorescent probe was used [30]. After loading the precursor DCFH_2_, the cells were exposed to an oxidative stress resulting in the formation of H_2_O_2_. Intracellular peroxidases then use this H_2_O_2_ to oxidize DCFH_2_ to the fluorescent DCF, antioxidants that intercept superoxide or other peroxide forming radicals in the cells increase the DCF fluorescence. ROS production decreased in the presence of EA having two *ortho* dihydroxy groups in L-6 myoblasts, even at very low concentrations. This showed that EA enters into the cells and maintains its high scavenging activity in the intracellular environment. It is pertinent to note that EAPA having two *ortho*-diacetoxy groups, also exhibited profound inhibition of ROS production in L-6 myoblasts as EA. The explanation is probably that, inside the cell, EAPA is rapidly de-esterified by the esterases, thus giving rise to the formation of the excellent scavenger, EA (Figure 2).

### 2.5. Inhibition of NADPH Catalysed Rat Liver Microsomal Lipid Peroxidation

**Figure 3:**
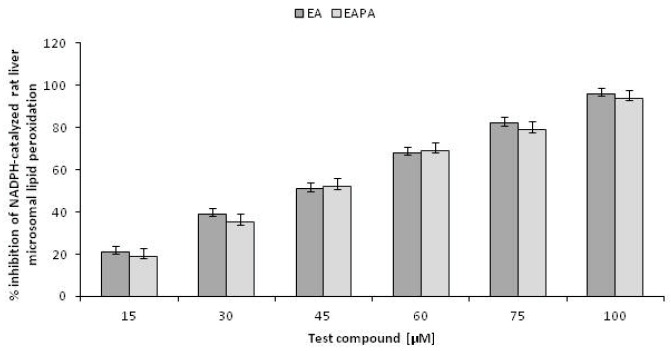
Effect of different concentration of EA and EAPA on NADPH-dependent lipid peroxidation in rat liver microsomes. Data represent mean ± S.D. from three independent experiments performed in triplicates, no significant differences were observed between EA and EAPA activities in all cases.

Lipid peroxidation processes play a central role in various diseases and also in the aging process due to its effects on cellular metabolism and functions. This is more complex and a number of modulatory effects are known which are important for maintaining cellular integrity and adaptive cellular responses. EA and EAPA produced inhibition of the initiation of lipid peroxidation in a concentration dependent manner (Figure 3). This result highlights the profound effectiveness of EA and EAPA in inhibiting the NADPH dependent lipid peroxidation in rat liver microsomes.

### 2.6. Influence of Different Concentration of EA and EAPA on A549 Cells

**Figure 4:**
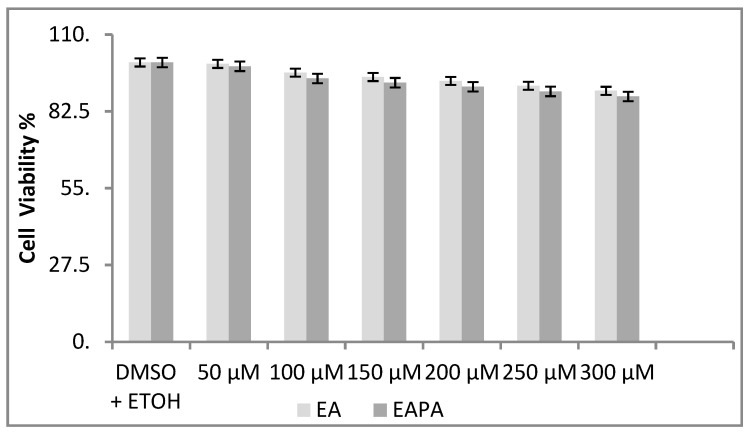
Influence of EA and EAPA on cell viability on A549 cells in a dose-dependent response by MTT assay. Cells were seeded into 96-well plates (1 × 10^4^ cells/well) and allowed to adhere for overnight. Cells were treated with increasing concentrations of EA and EAPA (50–300 μM) separately for 24 h; ethanol was used as control. Data represent mean of ± S.D. from three independent experiments and were performed in triplicates. *p* < 0.05.

The cytotoxic effects of EA and EAPA on A549 cells have been assessed using dose dependent experiments; the results clearly indicate that there was no apparent effect on cell viability of A549 cells, regardless of the dose of EA and EAPA (0–200 μM) even up to 24 h of exposure (Figure 4). However, there is a minor increase in cytotoxicity effects of EA and EAPA at concentrations of 250–300 μM. It is noteworthy to mention that EA and EAPA alone do not induce micronuclei formation in rat bone marrow and lung cells in-vivo experiments [26].

## 3. Materials and Methods

### 3.1. General Considerations

Ellagic acid, galvinoxyl, 2,2-diphenyl-1-picrylhydrazyl (DPPH) and cumene hydroperoxide were purchased from Sigma-Aldrich (St. Louis, MO, USA). 2′,7′-Dichlorodihydrofluorescein diacetate (DCFH_2_-DA) was obtained from Molecular Probes (Eugene, OR, USA). Dulbecco’s modified Eagle’s medium (DMEM), antibiotics, and sterile plastic ware for cell culture were procured from Flow Laboratory (Irvine, UK). Fetal bovine serum was acquired from GIBCO (Grand Island, NY, USA). NADPH, ADP and trichloroacetic acid (TCA) were obtained from Sisco Research Laboratory (Mumbai, India). Tris, FeCl_3_, thiobarbituric acid (TBA), dimethylsulphoxide (DMSO) of high purity were procured from local suppliers. Ellagic acid peracetate (EAPA) was synthesized and characterized at the Department of Chemistry of the University of Delhi, as previously described [26].

### 3.2. Electron Paramagnetic Resonance Spectroscopy

Stock solution of ellagic acid (10 mM) was prepared in ethanol (99%). Ellagic acid peracetate solution (10 mM) was prepared in a mixture of DMSO and ethanol (99%). Freshly prepared galvinoxyl radical solution (5 mM) in 99% ethanol was used for all the experiments. Systematic screening of EA and EAPA was done with free radicals, galvinoxyl and DPPH. The solutions were taken into glass capillaries, sealed, and measured using an ESP300 instrument (Bruker Spectrospin, Karlsruhe, Germany) equipped with a high sensitivity TM_110_ X-band cavity. Radical spectra were recorded at room temperature, using 0.6 G modulation, 1 mW microwave power, and a scan time of 42 s for a 30 G spectrum. Normally four spectra were accumulated for each measurement in order to obtain a suitable signal to noise ratio.

### 3.3. Spectrophotometric Analysis

Same Stock solutions of ellagic acid and ellagic acid peracetate were used for spectrophotometric analysis.

### 3.4. Cell Culture

L-6 cells from rat skeletal muscle were obtained from the American Type Culture Collection (Rockville, MD, USA). Cells were seeded in 75 cm^2^ flasks for tissue culture and grown in DMEM supplemented with 10% fetal bovine serum, 100 µg/mL streptomycin and 100 U/mL penicillin in an atmosphere of 5% CO_2_ at 37 °C. The cells reached confluency after 5 days (about 6 × 10^6^ cells) and were kept in culture as myoblasts by continuous passages at preconfluent stages, as previously reported [31].

### 3.5. Intracellular ROS Determination

DMEM was discarded and 5 mL of Phosphate Buffered Saline (PBS) containing 5.0 mM glucose were used to wash cells twice at 37 °C. Cells were gently scraped off with 5 mL PBS plus glucose at 37 °C and then the cell suspension added with 8–10 mL buffer solution; the contents were transferred to a centrifuge tube and centrifuged at 1200 rpm for 5 min (about 100× *g*). The supernatant was discarded and the pellet resuspended with a plastic Pasteur pipette in 5 mL PBS. After that pellet was resuspended and incubated with the probe. DCFH_2_–DA at a final concentration of 10 μM (from a stock solution of 10 mM in DMSO) was carried out for 30 min in the dark at 37 °C, as previously reported [30]. At the end of incubation, the cells were gently resuspended every 10 min; and were centrifuged at 1200 rpm for 5 min, the supernatant was discarded and the cell pellet was resuspended in 5 mL of PBS + glucose and centrifuged again. The last supernatant was discarded and the cell pellet resuspended in 2 mL PBS at a final concentration of 3 × 10^6^ cells/mL. Intracellular fluorescence was measured using Perkin–Elmer (Norwalk, CT, USA) LS 50B luminescence spectrometer under gentle magnetic stirring at 37 °C. Excitation (498 nm) and emission (530 nm) wavelengths were set using 5 and 10 nm slits, respectively for the two light paths. The assay was carried out in 3 mL cuvettes containing 200 μL cell suspensions in appropriate volume of buffer solution. Cumene hydroperoxide (diluted 1:100 in DMSO), was used as radical generator (final concentration 300 μM). DMSO at the concentrations used did not affect the fluorescence signal. The potency of antioxidant activity of the coumarin samples was determined by the decrease in the intracellular DCF fluorescence, reported as ΔF/10 min. This was calculated relative to the fluorescence change induced by 300 μM cumene hydroperoxide alone (100%). None of the compounds tested gave rise to fluorescence on their own.

### 3.6. Animals

Male albino rats of Wistar strain weighing 150–200 g were used for this study and supplied by Hindustan Lever Ltd., Mumbai (India). They were kept in climate-controlled animal quarters with water and food ad libitum in the animal house of V.P. Chest Institute, University of Delhi, India. This study was approved by Animal Research Committee of V. P. Chest Institute, University of Delhi, India. Procedures followed were in accordance with the ethical standards for the preparation of rat liver microsomes.

### 3.7. Preparation of Rat Liver Microsomes

Standardized methods were used for the preparing of rat liver microsomes for the study of lipid peroxidation [32]. In brief, rat liver (freshly excised) was suspended in 0.25 M sucrose solution. The solution was homogenized to obtain 30% homogenate, followed by centrifugation at 10,000× *g* for 30 min in a Sorvall refrigerated centrifuge. Then, the supernatant was spun at 100,000× *g* for 1 h in Beckman ultracentrifuge (model L) and pelleted microsomes was rinsed with 0.15 M KCl. This was resuspended in 0.15 M KCl and protein estimation was done using the well-known literature method [33].

### 3.8. Assay for Initiation of Lipid Peroxidation

A total of 2 mL of reaction mixture consisted of 0.025 M tris-HCI (pH 7.5), microsomes (1 mg protein), 3 mM ADP and 0.15 mM FeCl_3_, the samples were pre-incubated for 10 min at 37 °C, followed by the addition of the test compounds at a concentration of 10–100 µM in 0.2 mL of DMSO and then reincubated for 10 min at 37 °C. The reaction was started by the addition of 0.5 mM NADPH for initiation of enzymatic lipid peroxidation and further incubated for different intervals. The reaction was terminated by the addition of 0.2 mL of 50% TCA, followed by 0.2 mL of 5 N HCI and 1.6 mL of 30% TBA. The tubes were heated in an oil bath at 95 °C for 30 min, cooled and centrifuged at 3000 rpm. The intensity of the color of the thio-barbituric acid reactive substance (TBRS) formed was read at 535 nm [23,24].

### 3.9. Cell Viability Assay

Human lung adenocarcinoma cells (NSCLC, A549) were obtained from the National Center for Cell Science, Pune, India and maintained as per the method described by Goel et al., 2009 [34,35].

The cell viability was determined using the 3-(4,5-dimethyl thiazol-2yl)-2,5-diphenyltetrazolium bromide (MTT) assay. For this purpose, cells were grown in 96-well microtiter plates and treated with EA and EAPA separately at different concentrations (50–300 μM) in triplicates, for 24 h. Ethyl alcohol plus DMSO (100 μL) treated cells were taken as vehicle controls. In assay system, 100 μL MTT (5 mg/mL) was added to each well and incubated for 4 h at 37 °C in dark. Formazan crystals formed were dissolved in 100 μL ethyl alcohol plus DMSO and the absorbance was measured at 570 nm in an ELISA reader. Cell viability was calculated using the relationship:

%cell viability = (mean absorbance in test wells)/(mean absorbance in control well) × 100

### 3.10. Statistical Analysis

All assays were performed at least in triplicate and data were expressed as mean ± standard deviation. Statistical significance was determined with Student’s *t* test. Analysis of covariance and correlation were also performed for determining significance between different concentrations of EA and EAPA.

## 4. Conclusions

The results presented in this study mainly focus on the free radicals scavenging activity of EA and that of its acetylated product, EAPA in solvent and cellular systems. The effects are observed even at test compounds levels in the range from 1 to 10 µM, corresponding to the concentrations that would be relevant for therapeutic purposes. As expected, EA was an excellent radical scavenger but surprisingly, the corresponding acetylated product, EAPA also turned out to be good scavenger, even in the absence of an esterase. Our findings prove that EA and EAPA radical scavengers act like biological antioxidants and protect cells against oxidative stress.

## Figures and Tables

**Table 1:** Kinetics of the disappearance of EPR signal of DPPH due to reaction with EA and EAPA *^a^*.

Preincubation Time (min)	EA				EAPA			
	1 μM	2 μM	5 μM	10 μM	1 μM	2 μM	5 μM	10 yM
1	68 ± 4	54 ± 4	35 ± 5	0	74 ± 3	63 ± 3	48 ± 5	30 ± 6
5	63 ± 2	46 ± 2	15 ± 4	0	70 ± 2	59 ± 2	37 ± 4	22 ± 5
10	59 ± 3	42 ± 3	06 ± 3	0	66 ± 4	55 ± 3	21 ± 3	10 ± 3
30	54 ± 2	33 ± 4	0	0	63 ± 3	51 ± 2	02 ± 4	06 ± 2
60	49 ± 2	27 ± 2	0	0	58 ± 2	45 ± 2	0	02 ± 2
90	44 ± 3	21 ± 2	0	0	53 ± 4	41 ± 3	0	0
120	38 ± 4	15 ± 3	0	0	47 ± 5	36 ± 4	0	0
240	22 ± 3	09 ± 3	0	0	42 ± 3	31 ± 4	0	0

*^a^* Samples contained 100 μM DPPH and different concentrations of test compound in appropriate solvents. Data are shown as the percentage of radical remaining; 100% corresponds to 100 μM DPPH. Data shown are the mean ± S.D. of 4–6 individual experiments.

**Table 2:** Kinetics of decay of the galvinoxyl EPR signal remaining after incubation of 10 μM glavinoxyl with EA and EAPA *^a^*.

Preincubation Time (min)	EA				EAPA			
	1 μM	2.5 μM	5 μM	10 μM	1 μM	2.5 μM	5 μM	10 μM
1	69 ± 2	60 ± 4	35 ± 5	0	85 ± 6	74 ± 5	48 ± 6	30 ± 5
5	56 ± 4	37 ± 5	15 ± 3	0	82 ± 5	68 ± 3	37 ± 4	22 ± 3
10	44 ± 3	26 ± 3	06 ± 2	0	80 ± 3	53 ± 2	21 ± 2	10 ± 4
30	15 ± 4	0	0	0	69 ± 2	33 ± 4	11 ± 4	02± 2
60	09 ± 2	0	0	0	64 ± 4	21 ± 3	02 ± 3	0
120	0	0	0	0	57 ± 3	10 ± 2	0	0
180	0	0	0	0	49 ± 5	09 ± 2	0	0
240	0	0	0	0	43 ± 4	0	0	0
300	0	0	0	0	38 ± 3	0	0	0
360	0	0	0	0	29 ± 2	0	0	0

*^a^* Samples contained 10 μM galvinoxyl were separately incubated for different period of time at room temperature followed by measurement of decay of glavinoxyl EPR signal. Data are shown as the percentage of radical remaining; 100% corresponds to 10 μM galvinoxyl. Data shown are the mean ± S.D. of 4–6 individual experiments.

## Data Availability

The data that support the findings of this study can be obtained by contacting A.K. and/or S.I.
